# F-CphI represents a new homing endonuclease family using the Endo VII catalytic motif

**DOI:** 10.1186/s13100-018-0132-5

**Published:** 2018-08-09

**Authors:** Xiaoting Fang, YongLiang Jiang, Kim Li, Qinglu Zeng

**Affiliations:** 1 Department of Ocean Science, The Hong Kong University of Science and Technology, Clear Water Bay, Kowloon, Hong Kong, China; 2 School of Life Sciences, University of Science and Technology of China, Hefei, 230027 Anhui China; 3 Division of Life Science, The Hong Kong University of Science and Technology, Clear Water Bay, Kowloon, Hong Kong, China; 4 HKUST Shenzhen Research Institute, Shenzhen, China

**Keywords:** Homing endonuclease, Group I intron, F-CphI, Endonuclease VII, Bacteriophage

## Abstract

**Background:**

There are six known families of homing endonucleases, LAGLIDADG, GIY-YIG, HNH, His-Cys box, PD-(D/E)-XK, and EDxHD, which are characterized by their conserved residues. Previously, we discovered a novel homing endonuclease F-CphI encoded by ORF177 of cyanophage S-PM2. F-CphI does not resemble any characterized homing endonucleases. Instead, the C-terminus of F-CphI aligns well with the N-terminal catalytic domain of a Holliday junction DNA resolvase, phage T4 endonuclease VII (Endo VII).

**Results:**

A PSI-BLAST search resulted in a total of 313 Endo VII motif–containing sequences in sequenced genomes. Multiple sequence alignment showed that the catalytically important residues of T4 Endo VII were all well conserved in these proteins. Our site-directed mutagenesis studies further confirmed that the catalytically important residues of T4 Endo VII were also essential for F-CphI activity, and thus F-CphI might use a similar protein fold as Endo VII for DNA cleavage. A phylogenetic tree of the Endo VII motif–containing sequences showed that putative resolvases grouped into one clade while putative homing endonucleases and restriction endonucleases grouped into another clade.

**Conclusions:**

Based on the unique conserved residues, we proposed that F-CphI represents a new homing endonuclease family, which was named the DHHRN family. Our phylogenetic analysis could be used to predict the functions of many previously unknown proteins.

**Electronic supplementary material:**

The online version of this article (10.1186/s13100-018-0132-5) contains supplementary material, which is available to authorized users.

## Background

Many group I introns contain open reading frames (ORFs) that encode homing endonucleases. In a process called intron-homing, a homing endonuclease cleaves an intronless allele near the intron insertion site (IIS) and repair of the DNA break using the intron-containing allele as template transfers the intron and the homing endonuclease gene into the intronless allele [1]. Homing endonuclease genes are also found as optional free-standing genes inserted between two conserved genes. They can cleave one of the two conserved genes in genomes lacking the homing endonuclease gene. Analogous to intron-homing, repair of the DNA break transfers the homing endonuclease gene to the recipient genome, a process that has been called intronless homing [2, 3].

Homing endonucleases have been grouped into six families, LAGLIDADG, GIY-YIG, HNH, His-Cys box, PD-(D/E)-XK, and EDxHD, which were named for the conserved amino acid residues (reviewed by [4]). Crystal structures of the six homing endonuclease families have been determined [5–10]. Based on their structural similarities, it was suggested that the HNH and His-Cys box families should be combined to a ββα-metal superfamily [11]. The catalytic motifs of PD-(D/E)-XK and EDxHD families were also shown to be related [4].

Previously, we identified a novel homing endonuclease F-CphI [12]. It is encoded by ORF177 of cyanophage S-PM2 (genome accession # AJ630128.1), which is adjacent to the intron-containing *psbA* gene [13, 14]. While F-CphI has specificity for the homologous intronless *psbA* gene of phage S-BM4, the group I intron prevents F-CphI cleavage of the S-PM2 *psbA* gene [12]. In this novel arrangement, an intron prevents self-cleavage by a free-standing homing endonuclease, which in turn provides the intron with potential to invade intronless genomes. Thus, this process has been named “collaborative homing” [12].

**Fig. 1:**
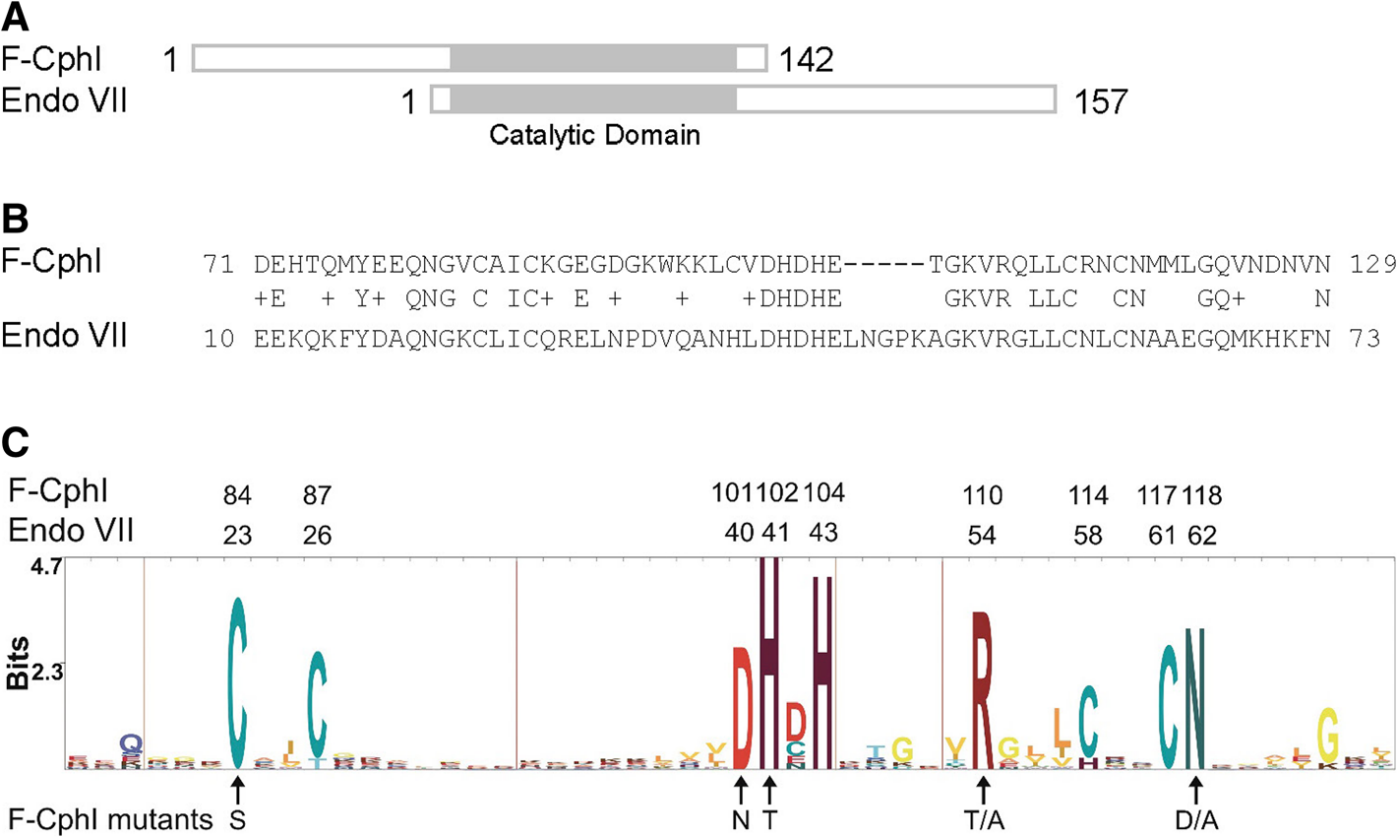
Sequence alignment. **a**, **b** The C-terminal part of F-CphI aligns with the N-terminal catalytic domain of Endo VII. **c** Sequence logo shows the consensus sequence from the multiple sequence alignment in Additional file 3: Figure S2. The positions of the conserved residues in F-CphI and Endo VII are shown on the top, and F-CphI mutants generated in this study are shown on the bottom. At each position of the sequence logo, the total height of a stack of letters shows the information content in bits that is calculated from a profile hidden Markov model (see Methods), and the height of a letter relative to the total height of letters at a position represents the letter’s frequency. The red lines indicate gaps in the multiple sequence alignment

ORF177 was originally annotated as similar to gene *49* of phage T4, which encodes endonuclease VII (Endo VII), a Holliday junction DNA resolvase [13, 14]. A BLAST search using F-CphI as the query sequence did not find any characterized homing endonuclease, but typically many other proteins annotated as “Endo VII”, “similar to Endo VII”, “resolvase”, and “gp 49”. Aside from Endo VII and its homologues in T-even phages, the enzymatic activities of these proteins had not been determined. Sequence alignment of F-CphI, Endo VII, and other Endo VII-like proteins showed a region of conserved sequence that included the catalytic domain of T4 Endo VII (Fig. 1a). Moreover, the catalytically important residues of Endo VII were all well conserved in these proteins. In this work, we conducted site directed mutagenesis on these conserved residues in F-CphI to determine whether they are functionally important. In addition, phylogenetic analysis allowed us to identify potential homing endonucleases and resolvases among the uncharacterized proteins. F-CphI appears to be the first characterized representative of a new family of homing endonucleases that use the Endo VII catalytic motif.

## Results

### Endonuclease assay with recombinant F-CphI

**Fig. 2:**
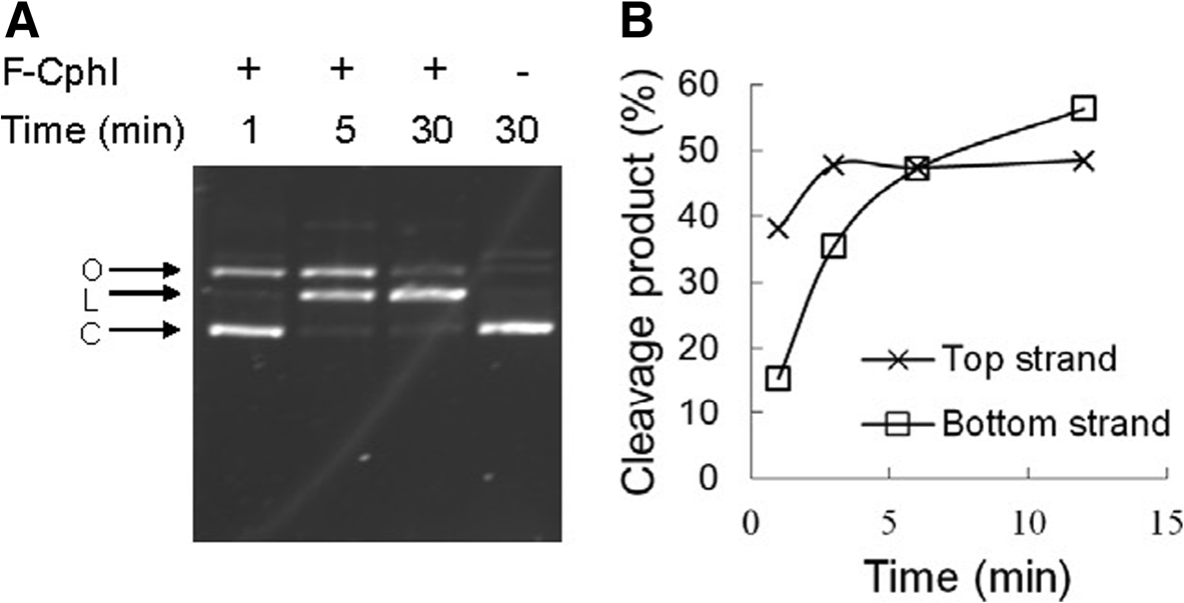
Strand preference of F-CphI. **a** Purified F-CphI (1600 nM) was used to digest plasmid DNA (5 nM) in the standard endonuclease assay buffer for different times**.** Closed-circular plasmid DNA (C) was nicked to form open circular DNA (O) and then nicked again on the other strand to form a linear product (L). **b** Time course activity assay on ^32^P-labeled oligonucleotide duplex. Oligonucleotides SBM4-60Top and SBM4-60Top-r were used to create a 60 bp duplex containing the F-CphI recognition site from the cyanophage S-BM4 *psbA* gene. The duplex was labeled on the top or the bottom strand by ^32^P. Labeled duplex substrates were digested by F-CphI and the cleavage products were separated by electrophoresis on a 4% denaturing polyacrylamide gel. Substrates labeled on the top or the bottom strand were examined separately. Percent cleavage products are shown over time

Previously we have used in vitro expressed F-CphI to map its cleavage site in the *psbA* gene of phage S-BM4 [12]. In order to further study the biochemical properties of this novel homing endonuclease, we cloned S-PM2 ORF177 into pBAD/*Myc*-HisB and induced His-tagged F-CphI expression in *Escherichia coli* cells. After large amounts of soluble F-CphI were induced, one step purification gave ~ 64% purity as observed on SDS-PAGE (Additional file 1: Figure S1A). Purified F-CphI was used to digest plasmid DNA containing the F-CphI recognition site for different times. As soon as F-CphI was added into the reaction, the closed-circular plasmid substrates were nicked into open-circular intermediate products, which were then converted into the final linear products (Fig. 2a). To know whether one DNA strand was preferentially nicked, top strand or bottom strand ^32^P labeled oligonucleotide duplexes containing the F-CphI recognition site were used as substrates. The top strand cleavage products appeared earlier than the bottom strand cleavage products, indicating that F-CphI preferred to nick the top strand first (Fig. 2b). Similarly, Endo VII [15] and the homing endonucleases I-SceI [16], I-TevI [17], I-TevII [18], and I-BmoI [19] also preferentially cleave one strand of the DNA substrate.

### Optimal conditions for F-CphI endonuclease activity

**Fig. 3:**
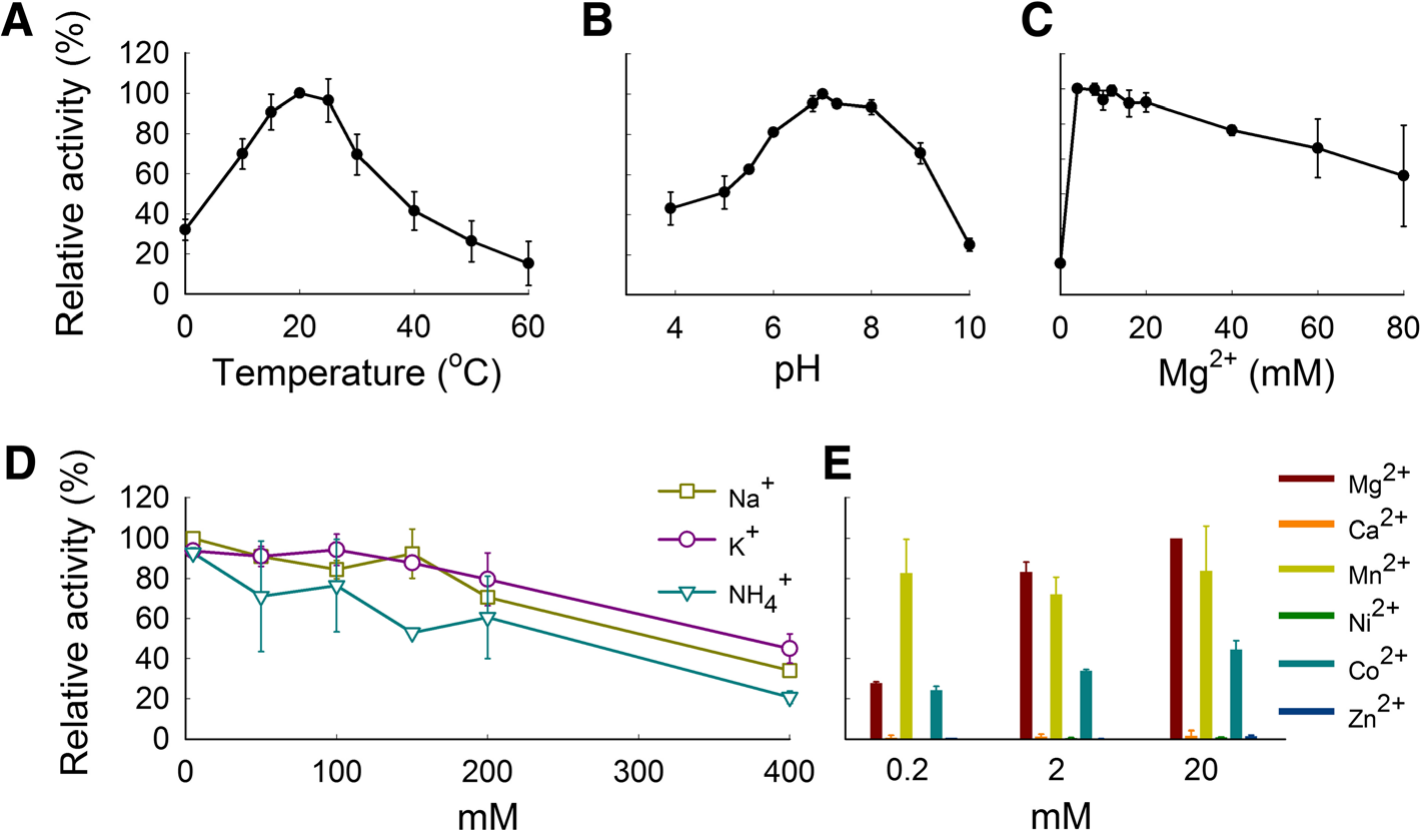
Optimization of conditions for DNA cleavage by F-CphI. Purified F-CphI (1600 nM) was used to digest plasmid DNA (5 nM) in different conditions for 30 min. The graphs show F-CphI endonuclease activity at different temperatures **a**, pH **b**, Mg^2+^ concentrations **c**, monovalent cation concentrations **d** and divalent cation concentrations **e** F-CphI endonuclease activity was calculated by expressing intensities of the linear products relative to the total plasmid DNA substrates. The highest activity for each variable was assigned a value of 100%. Error bars indicate the s.d. from two **b**, **d** and **e** or three **a** and **c** experiments

In order to determine the optimal conditions for F-CphI endonuclease activity on the plasmid DNA substrate, each parameter in the standard endonuclease assay was changed systematically (Fig. 3). F-CphI was most active at around 20 °C (Fig. 3a) and pH 7.0 (Fig. 3b), which is in contrast to 37–70 °C and alkaline pH for most biochemically characterized homing endonucleases [20] and T4 Endo VII [21]. The optimal temperature of F-CphI is consistent with the temperature of seawater where cyanophage S-PM2 was isolated, which encodes the gene for F-CphI. F-CphI activity showed a dependence on Mg^2+^, with an optimum of 4–20 mM (Fig. 3c). In addition, Mg^2+^ could be replaced by Mn^2+^ and Co^2+^, but not by Ca^2+^, Ni^2+^, or Zn^2+^ (Fig. 3e). Similar patterns of divalent cation dependence have previously been seen in the LAGLIDADG homing endonucleases I-DmoI [22], I-CreI [23], and the HNH homing endonuclease I-HmuI [7]. Endo VII activity was also dependent on Mg^2+^, which can be replaced by Mn^2+^ but not by Ca^2+^ [21]. Furthermore, F-CphI activity was not affected by a low concentration (5 mM) of monovalent ions (Na^+^, K^+^, or NH_4_^+^), but was inhibited at higher concentrations (Fig. 3d), a characteristic shared by homing endonucleases [24] and Endo VII [21].

### The catalytic domain of T4 endonuclease VII is conserved in F-CphI

The C-terminal part of F-CphI (residues 71–129) aligns with the N-terminal part of Endo VII (residues 10–73) (Fig. 1a and b), which is the catalytic domain of Endo VII [25, 26]. Using the putative C-terminal catalytic domain of F-CphI to do PSI-BLAST search, a total of 313 sequences were found in sequenced genomes (Additional file 2: Table S1). Multiple sequence alignment of these sequences (Additional file 3: Figure S2) revealed a highly conserved pattern of a central DHDH flanked by an N-terminal CX_2-4_C and a C-terminal CX_2_C (Fig. 1c). The first histidine in the DHDH region (H41 of Endo VII) was conserved in all the sequences (Additional file 3: Figure S2). The first aspartic acid (D40 of Endo VII) and the second histidine (H43 of Endo VII) were conserved in most of the sequences (Additional file 3: Figure S2). The arginine residue (R54 of Endo VII) after the DHDH sequence was conserved in all the sequences (Fig. 1c, Additional file 3: Figure S2). The asparagine (N62 of Endo VII) distal to CX_2_C was conserved in most sequences (replaced by R in one sequence Strvi_0243) (Fig. 1c, Additional file 3: Figure S2).

The catalytic domain of T4 Endo VII has been studied extensively. The CX_2-4_C and CX_2_C sequences have been shown to coordinate one atom of zinc in Endo VII [27]. D40 and N62 are the metal ion binding residues in the active site of T4 Endo VII [25, 28]. H41 and H43 are catalytically important in Endo VII [25, 26]. Although the second aspartic acid (D42) in the DHDH region was conserved in many sequences, it is not essential for Endo VII [29]. R54 was shown to be within the active site of T4 Endo VII and near the metal ion [26], but its function is still unknown. The fact that the catalytically important residues for Endo VII were all conserved in F-CphI (Fig. 1b) suggested to us that they may play similar roles in F-CphI.

### Site-directed mutagenesis confirms that the conserved Endo VII residues are also essential for F-CphI

Site-directed mutagenesis has been done on T4 Endo VII. C23S and C61S mutants were inactive and they cannot bind to the DNA substrate [27]. C26S and C58S mutants were still active, but when both cysteines were mutated the mutant was inactive and failed to bind to the DNA substrate [27]. D40N, D40A, H41T, H43T [29], and N62D [26] were inactive but they can bind to the DNA substrate, suggesting that these residues are important for catalytic activity. To test whether the essential residues of Endo VII are also important for F-CphI activity, we carried out site-directed mutagenesis on F-CphI at the corresponding residues to Endo VII (C84S, D101N, H102T, N118A, and N118D). No mutation had been made on the conserved arginine of T4 Endo VII, but since it is highly conserved and is near other catalytically important residues, the arginine on F-CphI was also mutated (R110A and R110T) to explore its function.

**Fig. 4:**
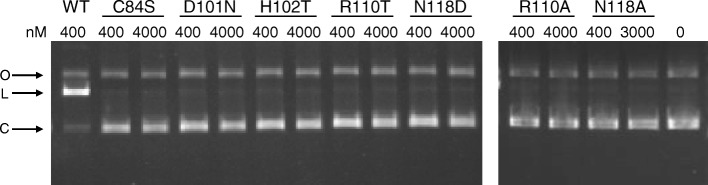
Activity comparison of F-CphI wild type and mutants. F-CphI wild type (400 nM) and mutants (400 nM and 3000 nM for N118A; 400 nM and 4000 nM for others) were incubated with 5 nM closed-circular plasmid DNA (C) at 25 °C for 1 h. For N118A, 4000 nM cannot be used since it bound to the DNA substrate non-specifically and showed a smear in the gel (data not shown). Reaction products were separated on a 0.8% agarose gel. Positions of linear (L) and open circular (O) products are shown. DNA substrate without protein added was run in the last lane

After expression and purification, the F-CphI mutants had similar soluble protein yields as that of the wild type (Additional file 1: Figure S1B), suggesting that mutations may not disrupt the general folding of the protein. A plasmid containing the F-CphI recognition site was used to conduct endonuclease assays to compare the activities of F-CphI wild type and mutants. The closed-circular plasmid DNA was linearized by 400 nM wild type protein, however, cleavage products were not obviously seen for all the mutants, even at ~ 10-fold excess protein concentration (Fig. 4). To analyze the cause for abolished DNA cleavage observed with the F-CphI mutants, the DNA binding property was assayed by electrophoretic mobility shift assay using a ^32^P-labeled 60-bp oligonucleotide containing the F-CphI recognition site. Among the mutant proteins, only D101N and H102T can bind DNA (Additional file 4: Figure S3A), but their DNA binding affinities were lower than that of the wild type as shown by their higher Kd values (Additional file 4: Figure S3C). Using an unlabeled non-specific 41-bp DNA complex as the competitor, competition assays confirmed that the binding of the wild type, D101N and H102T to DNA substrate was specific (Additional file 4: Figure S3B). In summary, similar to T4 Endo VII [29], our mutagenesis studies suggested that C84 of F-CphI is important for DNA binding, while D101 and H102 are essential for DNA cleavage. The difference is that F-CphI mutant N118D cannot bind to DNA, while the corresponding Endo VII mutant N62D can bind to DNA [26]. In addition, our results showed that R110 of F-CphI is an essential residue for DNA binding, but it is not clear whether it is also essential for DNA cleavage (see Discussion).

### Phylogenetic analysis of proteins containing the Endo VII motif

**Fig. 5:**
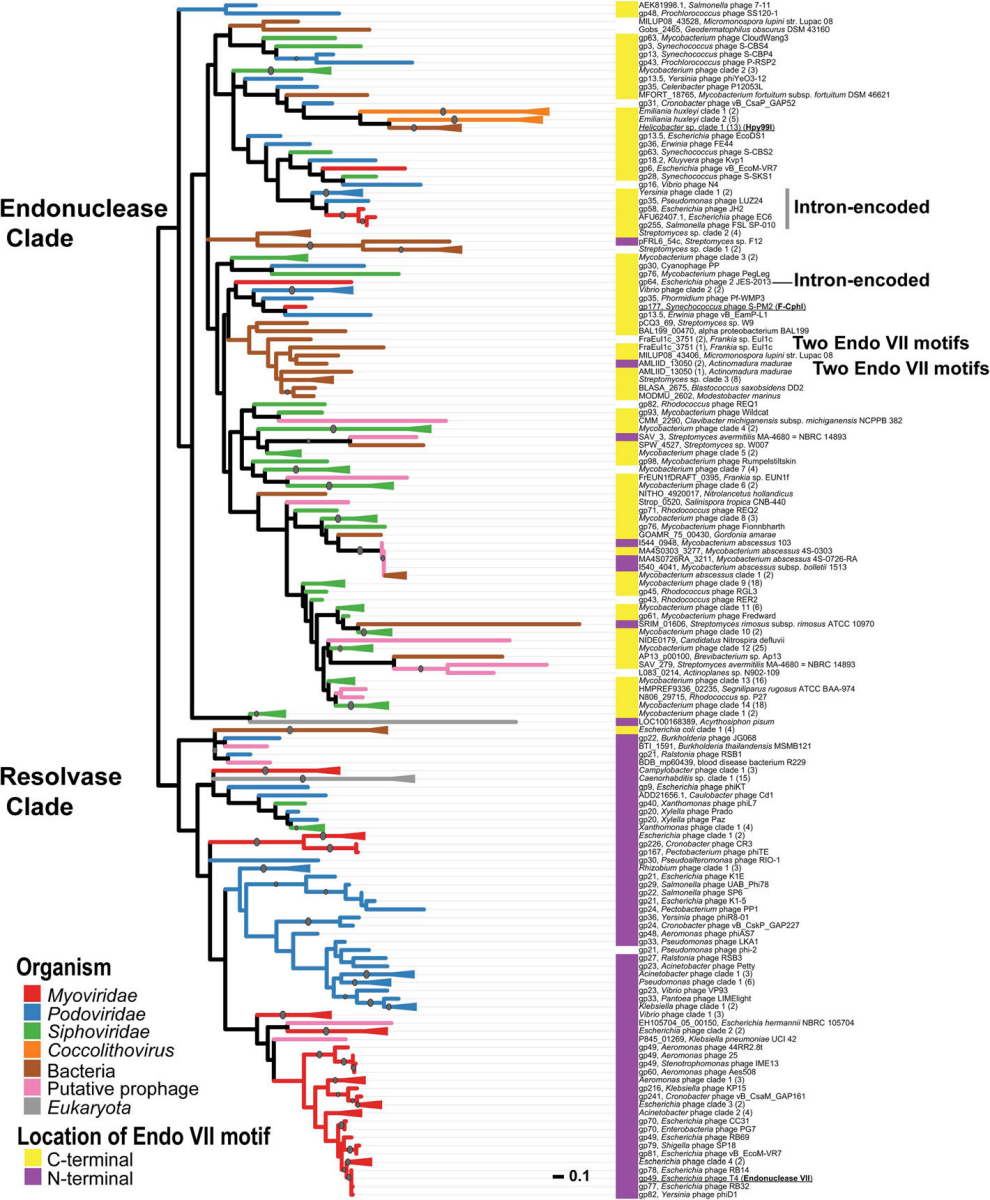
Phylogenetic tree of the Endo VII motif–containing sequences. An unrooted radial tree was drawn, as there was not a suitable outgroup. The top portion of the tree was named Endonuclease Clade and the bottom portion was named Resolvase Clade. The branch length represents the expected number of substitutions per site, and the scale bar shows 0.1 substitution per site. The branch color shows the organism type. A color bar on the left of a sequence name indicates the location of the Endo VII motif in that sequence, yellow for C-terminal and purple for N-terminal. Dots on the branches indicate bootstrap values > 50%. Branches with triangles on the end contain more than one sequences from the same organism type whose Endo VII motifs are in similar locations of a protein. The number in the parenthesis after a clade name indicates the number of sequences in that clade and the sequences are listed in Additional file 7: Table S2

Based on the multiple sequence alignment (Additional file 3: Figure S2), we built a phylogenetic tree using the protein sequences that contain the Endo VII motif. The phylogenetic tree consisted of two major branches (Fig. 5). The bottom portion of the tree contained T4 Endo VII (sequence name underlined) and sequences that aligned their whole length with Endo VII (Fig. 5, Additional file 2: Table S1). Moreover, most sequences in the bottom portion of the tree had the Endo VII motif on their N-termini (Fig. 5), which is similar to the domain arrangement of T4 Endo VII. The top portion of the tree contained F-CphI (sequence name underlined) and proteins that aligned the whole length with F-CphI (Fig. 5, Additional file 2: Table S1). Interestingly, most sequences in the top portion of the tree had the Endo VII motif on their C-termini (Fig. 5), similar to the domain arrangement of F-CphI. Our further analysis shown below suggested that sequences in the bottom portion of the tree might be resolvases and those in the top portion might be endonucleases, and hence we named them resolvase and endonuclease clades, respectively.

In the resolvase clade, sequences from myoviruses including T4 Endo VII clustered together (Fig. 5) and they are likely to be the Holliday junction resolvases of these phages. Many sequences from podoviruses also clustered together (Fig. 5). Interestingly, while phage T7 uses endonuclease I as its Holliday junction resolvase, none of these podoviruses encodes an endonuclease I homolog. Therefore, these podoviruses might use Endo VII as their Holliday junction resolvase. The resolvase clade also contained four sequences from putative prophage fragments in bacterial genomes (BTI_1591, BDB_mp60439, EH105704_05_00150, P845_01269) (Fig. 5). In these bacterial genomes, the Endo VII motif-containing sequences were often adjacent to integrase genes, portal protein genes, or putative phage genes, and thus they were probably resolvases of prophages. Furthermore, we identified 15 sequences from *Caenorhabditis* (Fig. 5) that were annotated as DNA polymerase B. These proteins are of different lengths, and the Endo VII motifs are in different positions in these proteins (Additional file 2: Table S1). The function of the Endo VII motif in DNA polymerase B remained unknown.

In the endonuclease clade, the podoviruses (Fig. 5) all encode the T7 resolvase endonuclease I, and thus the Endo VII motif–containing proteins might not be resolvases. Moreover, sequences from *Yersinia* phage Berlin, *Yersinia* phage YpP-G, and *Pseudomonas* phage LUZ24 are embedded in a group I intron in the DNA polymerase gene (Intron-encoded, Fig. 5), indicating that they are homing endonucleases [30]. Self-splicing group I introns have also been identified in the DNA polymerase genes of several T7-like phages [31, 32]. There are six sequences from myoviruses (Fig. 5). Four of them are intron-encoded (Fig. 5, sequences from *Escherichia* phage JH2, *Escherichia* phage EC6, *Salmonella* phage FSL SP − 010, and *Escherichia* phage 2 JES − 2013), while F-CphI is associated with a group I intron. The only sequence that is not encoded/associated with an intron is gp6 from *Escherichia* phage vB EcoM−VR7. Since this phage has another Endo VII motif-containing protein (gp81) in the resolvase clade, gp6 is probably a free-standing homing endonuclease. There are 13 sequences from *Helicobacter* and one of them Hpy99I has been shown to be a type II restriction endonuclease [33]. Similar to Hpy99I, 12 out of the 13 *Helicobacter* sequences (except for EMH02975) are adjacent to a methylase, which protects bacterial genomes from self-cleavage. Thus, these sequences are likely to be restriction endonucleases.

## Discussion

Multiple sequence alignment of F-CphI, T4 Endo VII, and the other Endo VII motif–containing proteins revealed a different consensus sequence from that of the HNH family endonucleases (Additional file 5: Figure S4). The HNH family was named based on a conserved pattern of a central asparagine flanked by two histidines at some distance (the second histidine is often replaced by an asparagine) (Additional file 5: Figure S4) [34, 35]. Superposition of the catalytic domains of T4 Endo VII and the HNH homing endonuclease I-HmuI showed that H41 and N62 of Endo VII correspond to the first and the second histidines of the HNH motif in I-HmuI, respectively [7, 25]. However, Endo VII and F-CphI do not contain the central asparagine that is conserved in the HNH family endonucleases, and they both contain several additional conserved residues (D40, H43, and R54 in Endo VII) (Additional file 5: Figure S4). The consensus sequence of the Endo VII motif–containing proteins are also different from that of the His-Cys box family homing endonucleases (Additional file 5: Figure S4). Therefore, we proposed that F-CphI represents a new homing endonuclease family and we named it the DHHRN family based on the conserved residues.

**Fig. 6:**
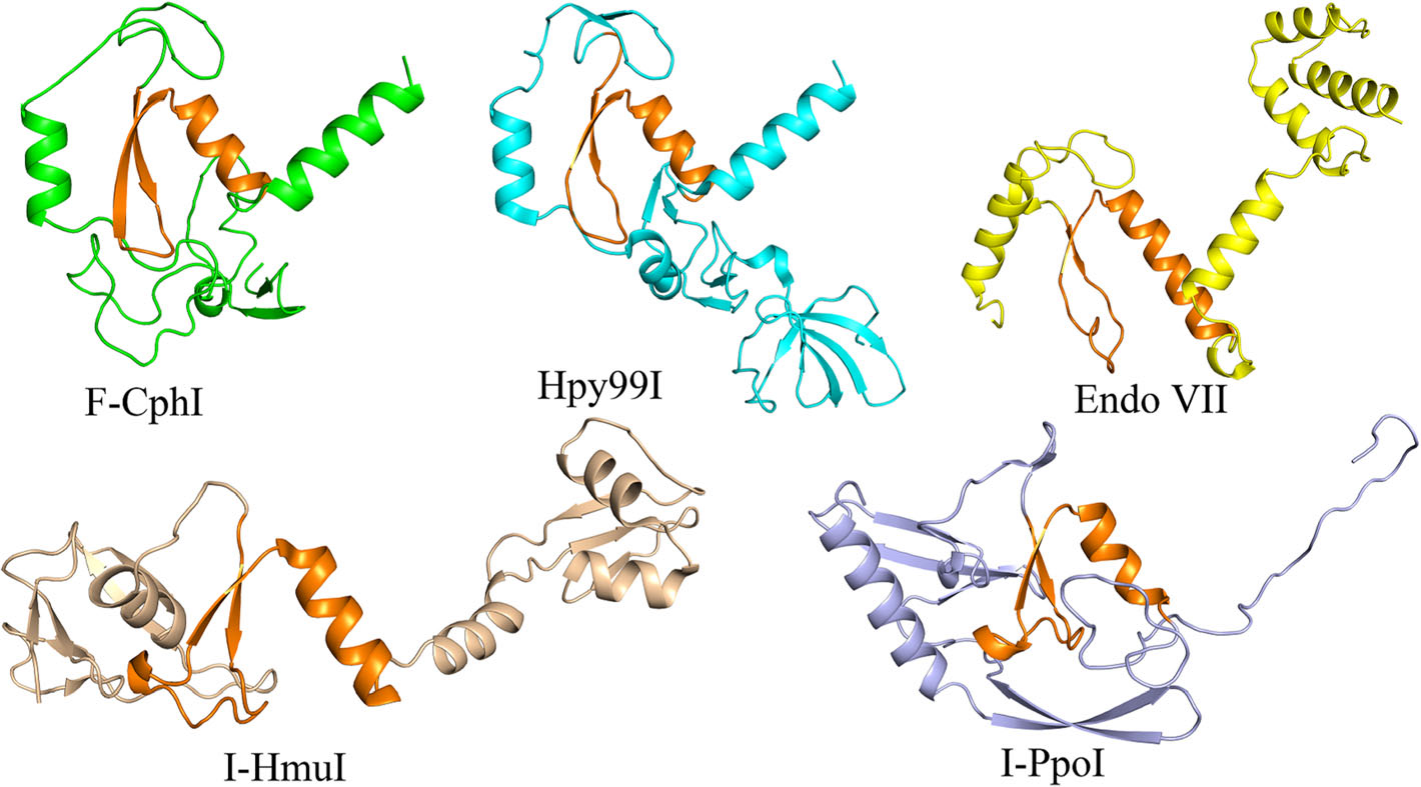
Structures of the ββα-metal superfamily endonucleases. Protein structures of Hpy99I [36], Endo VII [26], I-HmuI [7], and I-PpoI [5] were obtained from previous studies. The structure model of F-CphI was generated based on Hpy99I structure using the online tool SWISS-MODEL (www.swissmodel.expasy.org/). The conserved active-site motif of each protein was colored orange and presented in the same orientation

Among the DHHRN endonucleases, the crystal structures of T4 Endo VII [28] and Hpy99I [36] have been solved together with their DNA substrates. Although Endo VII is a Holliday junction DNA resolvase and Hpy99I is a restriction endonuclease, their endonuclease domain structures are almost identical and are also similar to those of the HNH family homing endonuclease I-HmuI and the His-Cys box family homing endonuclease I-PpoI (Fig. 6) [36]. Together with the HNH and His-Cys box families [11], the DHHRN family also belongs to the ββα-metal endonuclease superfamily.

Our mutagenesis experiments suggested that D101 and H102 were catalytically important for F-CphI. The corresponding residues are not only conserved in Endo VII and Hpy99I, they are also within the active site and have catalytic functions [28, 36]. Thus, the active site structure of F-CphI is likely to be similar to those of Endo VII and Hpy99I. Indeed, we used Hpy99I structure to model the structure of F-CphI and found that their core structures harboring the active site are almost identical (Fig. 6). Since Hpy99I uses its active site region for both DNA cleavage and binding [36], F-CphI may also use its active site region for DNA binding. Furthermore, our mutagenesis experiments showed that R110 and N118 affected DNA binding of F-CphI, while the corresponding residues are shown in Hpy99I to have catalytic functions [36]. Thus, we proposed that R110 and N118 of F-CphI may play a role in both DNA binding and cleavage.

It has been shown that the catalytic motifs of LAGLIDADG, GIY-YIG, HNH, His-Cys box, PD-(D/E)-XK, and EDxHD homing endonucleases are used by proteins of a variety of functions. These proteins include non-specific DNA-degradation colicins, restriction endonucleases, DNA repair enzymes, Holliday junction resolvases, intron-splicing factors, and transcription factors [4]. It is still not clear whether these proteins diverged from a common ancestor or the homing endonucleases were modified by the host organisms to be specialized in other cellular functions [4]. Similar to the six established homing endonuclease families, our phylogenetic analysis (Fig. 5) showed that the DHHRN motif can be used by homing endonucleases, Holliday junction resolvases, restriction endonucleases, and possibly DNA polymerases. Our analysis facilitates the functional prediction of many previously unknown proteins.

## Conclusions

The catalytically important residues of T4 Endo VII are all conserved and essential for F-CphI activity. F-CphI represents a new homing endonuclease family, the DHHRN family. Our phylogenetic analysis showed that the DHHRN family proteins include homing endonucleases, Holliday junction resolvases, restriction endonucleases, and possibly DNA polymerases.

## Methods

### Cloning of F-CphI gene and site-directed mutagenesis

F-CphI wild type and mutants were cloned into pBAD/*Myc*-HisB plasmid (Invitrogen), in which protein expression is tightly controlled by the P_BAD_ promoter and can be induced by L-arabinose. Primers CyaU-Nco and CyaD-Xba were used to amplify the F-CphI gene from S-PM2 phage. PCR was carried out with 0.02 U/μl KOD HiFi DNA polymerase (Novagen) in 120 mM Tris-HCl (pH 8.0), 10 mM KCl, 6 mM (NH_4_)_2_SO_4_, 0.1% Triton X-100, 0.001% BSA, 1 mM MgCl_2_, 0.2 mM dNTPs, and 0.4 μM of each primer. PCR cycling conditions consisted of a hot start at 94 °C for 5 min, followed by 25 cycles (98 °C for 15 s, 50 °C for 2 s and 74 °C for 20 s), followed by incubation at 74 °C for 7 min. Site-directed mutagenesis was performed by a PCR based overlap extension method [37]. PCR products were inserted into the NcoI and XbaI sites of the pBAD/*Myc*-HisB vector and transformed into *E. coli* Top10 cells. Primer CyaD-Xba does not have a stop codon and hence the cloned genes are in frame with the downstream His-tag. The desired mutations were confirmed by sequencing. Oligonucleotides are listed in Additional file 6: Table S3.

### Protein expression and purification

Strains for expressing F-CphI wild type and mutants were grown at 37 °C overnight in the LB medium containing 50 μg/ml ampicillin. Overnight cultures were diluted 1:100 with the same medium, grown at 37 °C until OD_600_ = 0.5, and then used for protein induction. The concentration of inducer (L-arabinose), the induction time, and temperature were optimized. Maximum protein expression was induced by 0.02% L-arabinose (final concentration) at 16 °C for 24 h. Bacterial cells were harvested by centrifugation at 6000 g for 20 min. The cell pellet was suspended with the binding buffer (20 mM sodium phosphate, 500 mM NaCl, 20 mM imidazole, pH 7.4) and disrupted by sonication. The crude lysate was centrifuged at 10,000 g for 20 min and the supernate was loaded on a HisTrap FF crude column (GE Healthcare). Protein purification was performed according to the manufacturer’s instructions. Protein was eluted from the column with the elution buffer (20 mM sodium phosphate, 500 mM NaCl, 500 mM imidazole, pH 7.4) and stored in 50% glycerol at − 20 °C.

### Endonuclease assay with plasmid substrate

The plasmid pSBM4-TA [12] containing the phage S-BM4 *psbA* gene was used as substrate for F-CphI. 5 nM plasmid was incubated with 1600 nM purified F-CphI in the standard endonuclease assay buffer (50 mM Tris-HCl, pH 7.5, 50 mM NaCl, 10 mM MgCl_2_ and 0.1 mg/ml BSA) at 25 °C for 30 min when the reaction was still linear. The reaction was stopped by adding 1 μl 10× endonuclease stop buffer (100 mM Tris-HCl, 25 mM EDTA, 5% SDS, pH 7.5) and kept on ice. Reaction products were separated in a 0.8% agarose gel and stained with EtBr. To determine the optimal conditions for F-CphI, each parameter in the standard endonuclease buffer was changed while others remained the same. Formate (for pH 3.9), succinic acid (for pH 5.0, 5.4 and 6.0), PIPES (for pH 6.8, 7.0 and 7.3), Tris (for pH 8.0), and CHES (for pH 9.0 and 10.0) were used to prepare different pH buffers and their final concentrations were all 50 mM. The optimal endonuclease assay buffer was characterized as 50 mM PIPES (pH 7.0), 5 mM MgCl_2_, 50 mM KCl and 0.1 mg/ml BSA. To compare the activities of F-CphI wild type and mutants, 5 nM pSBM4-TA plasmid was incubated with different amounts of proteins at 25 °C for 1 h in the optimal endonuclease assay buffer.

### Endonuclease assay with ^32^P-labeled substrate

Oligonucleotide duplex containing the F-CphI cleavage site [12] was generated by annealing complementary oligonucleotides SBM4-60Top and SBM4-60Top-r at 90 °C for 5 min and cooling to 55 °C in 25 mM Tris/HCl (pH 8.0) and 50 mM NaCl. Individually 5′ end-labeled targets were generated by labeling one of the oligonucleotides on its 5′ end (with [γ-^32^P]ATP and T4 polynucleotide kinase) before being used in annealing reactions with an unlabeled partner. Labeled duplexes were purified using a QIAquick PCR Purification Kit (Qiagen). 10 nM labeled duplex was incubated with 1 μM F-CphI in the optimal endonuclease assay buffer at 25 °C. Reaction products were extracted with an equal volume of phenol and separated on a 4% denaturing polyacrylamide gel. Sequences of SBM4-60Top and SBM4-60Top-r are listed in Additional file 6: Table S3.

### Electrophoretic mobility shift assay

To compare the DNA binding ability of F-CphI wild type and mutants, both specific and nonspecific DNA duplexes were used. A specific oligonucleotide duplex containing the F-CphI cleavage site was generated by annealing 5′ end labeled SBM4-60Top and unlabeled SBM4-60Top-r. Nonspecific competitor duplex was generated using oligonucleotide endoV41A and its complementary sequence endoV41comp (Additional file 6: Table S3). For assays without competitor, 2 nM labeled SBM4-60Top duplex was incubated on ice for 15 min with different amounts of protein in 10 mM Tris-HCl (pH 8.0), 2% glycerol, 2 μg/ml poly-dIdC, 2 μg/ml BSA and 0.2 mM DTT in total 20 μl reaction volume. For assays with nonspecific competitor, 2 nM labeled SBM4-60Top duplex and 200 nM protein were incubated with different amounts of endoV41A duplex. The free DNA and protein-bound complexes were separated on 8% native polyacrylamide gel with 1X TBE buffer (89 mM Tris, 2 mM EDTA, 89 mM Boric acid, pH 8.3).

### Binding affinities of F-CphI wild type and mutants to DNA substrates

Quantitation of band intensities from the electrophoretic mobility shift assay were performed in the GelQuant.NET software (biochemlabsolutions.com). Band intensity of DNA-bound complex at protein concentration of 0 nM was used as the background signal, and was subtracted from the signal intensities obtained from all the bands. The fraction of DNA bound was thus calculated from bound/(bound + unbound), and was plotted versus the concentrations of F-CphI wild type, and D101N and H102T mutants, respectively. The data were then fitted by non-linear regression model with the nlsLM function of minpack. lm package in R, using the following equation modified from previous studies [38, 39]:

$$ \mathrm{F}=\mathrm{A}\times {\mathrm{P}}_0/\left(\mathrm{Kd}+{\mathrm{P}}_0\right) $$

where F is the fraction of DNA bound, A is the maximal fraction of DNA bound, P_0_ is the concentration of total protein, and Kd is the apparent equilibrium dissociation constant.

### Homolog search

In-house BLAST searches were carried out against the downloaded non-redundant (nr) protein database (http://www.ncbi.nlm.nih.gov/BLAST/). In each search, we performed BLASTp and PSI-BLAST with two iterations and set a final *e*-value cut-off of 1E-4. In search of the Endo VII homologs, both the catalytic domain of T4 Endo VII (residues 1–97) and the corresponding sequence of F-CphI (residues 62–139) were used as the query protein sequences. The resulting 313 protein sequences from complete genomes were fetched from NCBI for further analysis. In search of the HNH and His-Cys box family proteins, the catalytic domains of I-HmuI (residues 49–97) from *Bacillus* phage SP01 and I-PpoI (residues 93–127) from *Physarum polycephalum* were used as the query sequences, respectively. Sequences from incomplete genomes or obtained from environmental samples were discarded prior to protein sequence extraction.

### Multiple sequence alignment

MAFFT [40] and Clustal X [41] were used for multiple sequence alignment of the homologous sequences. The resulting alignment was then manually refined through editing in Alignment Explorer of MEGA6 [42]. The final alignment, 133 bp in length with gaps, represented the most conserved sequence blocks across all compared organisms. The alignment was then analyzed on ESPript (http://espript.ibcp.fr/) to obtain the consensus sequence with conserved amino acids highlighted.

### Sequence logo

In order to profile the consensus sequence from the multiple sequence alignment, we generated sequence logo on the Skylign website (http://skylign.org), which is based on the profile hidden Markov model (HMM) analysis [43]. Profile HMM establishes a position-specific scoring sequence profile representing a multiple sequence alignment [43]. Skylign creates a graphical sequence logo (HMM logo) from the sequence alignment, which not only represents the extent of conservation at each position, but also shows the probabilities of a profile HMM [44]. In the HMM logo, the height of a stack of letters at each position shows the information content in bits, which represents the extent to which the position-specific distribution of letters differs from that of the background. At each position, the height of a letter relative to the total height of the stack of letters correlates to a particular letter’s frequency at that position [44].

### Phylogenetic analysis

ProtTest [45] was used with default parameters to select the best amino acid substitution model. PhyML [46] was used for phylogenetic analysis of the Endo VII domain homologs, based on the maximum-likelihood principle. The parameters for the command-line PhyML program were set as: -d aa -b 100 -m Blosum62 -f m -v e -s SPR -o tlr. The output phylogenetic tree was demonstrated via iTOL (http://itol.embl.de).

## Additional files

Additional file 1: **Figure S1.** Purification of F-CphI wild type and mutants. (**A**) *E. coli* cells were induced to express F-CphI and then disrupted by sonication. The crude lysate was centrifuged and the supernate was loaded on a HisTrap FF crude column. Five elutions (1 ml each) were collected. The crude lysate, supernate, pellet, and five elutions were separated on 15% SDS-PAGE. (**B**) Purified F-CphI wild type and mutants (second elutions) were separated on 15% SDS-PAGE. (PDF 233 kb)

Additional file 2: **Table S1.** Endo VII motif-containing proteins. Proteins containing the Endo VII motif were revealed by PSI-BLAST searches. In the last column, the position of the Endo VII motif in a protein is shown. The Endo VII motif of a protein could be in the C terminus (C), the N terminus (N), or in the middle (indicated by residue numbers/full length of a protein). In the same column, F-CphI and Endo VII indicate that a protein aligns with the full length of F-CphI or Endo VII, respectively. (XLSX 29 kb)

Additional file 3: **Figure S2.** Multiple sequence alignment of the Endo VII motif sequences. Dots represent gaps in the alignment. Totally conserved residues at a position are shown by white letters in red background. Residues with conservation above 70% at a position are shown by red letters in blue boxes. The position numbers of F-CphI and Endo VII residues are shown on top of the alignment. (PDF 1993 kb)

Additional file 4: **Figure S3.** Gel shift assay of F-CphI wild type and mutants. (A) 2 nM ^32^P labeled 60 bp duplex containing the F-CphI recognition site was incubated on ice for 15 min with increasing concentration of each protein. The free DNA (F) and protein-bound complexes (C) were separated on 8% native polyacrylamide gel. (**B**) 2 nM ^32^P labeled 60 bp duplex containing the F-CphI recognition site and 200 nM protein were incubated with increasing concentration of unlabeled non-specific 60 bp duplex (0 nM to 20 nM). The first lane in each gel shows the pattern of free DNA (no protein was added in the reaction). (**C**) Using the gels in **A**, the fractions of protein-bound complexes for wild type, D101N, and H102T were plotted against protein concentrations. The binding curves were generated from the non-linear regression fitted data, and were used to estimate the apparent equilibrium dissociation constant (Kd). Kd values are shown in each graph and errors represent 95% confidence interval. (PDF 126 kb)

Additional file 5: **Figure S4.** Sequence logos of the DHHRN, HNH, and His-Cys box endonuclease families. Sequences near the active site of an endonuclease family are used to generate sequence logos. At each position of the sequence logo, the total height of a stack of letters shows the information content in bits that is calculated from a profile hidden Markov model, and the height of a letter relative to the total height of letters at a position represents the letter’s frequency. The red lines indicate gaps in the multiple sequence alignment. Above each sequence logo, the corresponding residue numbers of Endo VII, I-HmuI, and I-PpoI are shown, which are representatives of the DHHRN, HNH, and His-Cys box families, respectively. Black boxes show the corresponding catalytic residues used by Endo VII, I-HmuI, and I-PpoI. (PDF 351 kb)

Additional file 6: **Table S3.** Oligonucleotides used in this study (restriction sites are underlined and mutated sites are italicized). (DOCX 12 kb)

Additional file 7: **Table S2.** Clades of Endo VII motif-containing proteins as shown in Fig. 5. (XLSX 17 kb)

## Abbreviations

Endo VII: Endonuclease VII
IIS: Intron insertion site
ORF: Open reading frames
