# Supplementary material for: F-CphI represents a new homing endonuclease family using the Endo VII catalytic motif
Source: Mob DNA. 2018 Aug 9;9:27. doi: 10.1186/s13100-018-0132-5 (PMC6083498; doi:10.1186/s13100-018-0132-5)
Supplement: Supplementary file 1 — Figure S1. Purification of F-CphI wild type and mutants. (A) E. coli cells were induced to express F-CphI and then disrupted by sonication. The crude lysate was centrifuged and the supernate was loaded on a HisTrap FF crude column. Five elutions (1 ml each) were collected. The crude lysate, supernate, pellet, and five elutions were separated on 15% SDS-PAGE. (B) Purified F-CphI wild type and mutants (second elutions) were separated on 15% SDS-PAGE. (PDF 233 kb) [file 13100_2018_132_MOESM1_ESM.pdf]

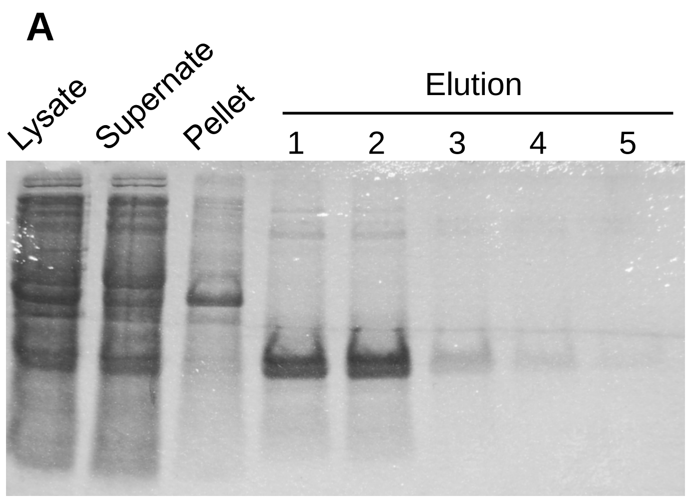

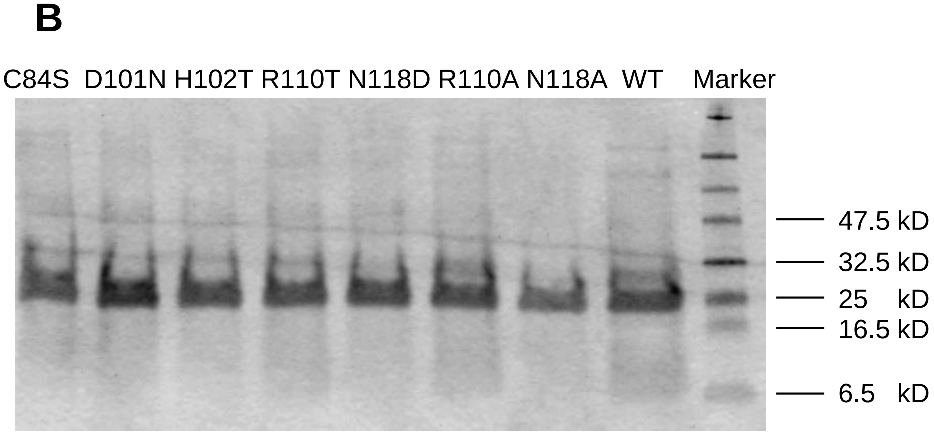

Supplementary Figure 1. Purification of F-CphI wild type and mutants

(A) *E. coli* cells were induced to express F-CphI and then disrupted by sonication. The crude lysate was centrifuged and the supernate was loaded on a HisTrap FF crude column. Five elutions (1 ml each) were collected. The crude lysate, supernate, pellet, and five elutions were separated on 15% SDS-PAGE. (**B**) Purified F-CphI wild type and mutants (second elutions) were separated on 15% SDS-PAGE.
