# Supplementary material for: F-CphI represents a new homing endonuclease family using the Endo VII catalytic motif
Source: Mob DNA. 2018 Aug 9;9:27. doi: 10.1186/s13100-018-0132-5 (PMC6083498; doi:10.1186/s13100-018-0132-5)

Supplementary Figure 2. Multiple sequence alignment of the Endo VII motif sequences

Dots represent gaps in the alignment. Totally conserved residues at a position are shown by white letters in red background. Residues with conservation above 70% at a position are shown by red letters in blue boxes. The position numbers of F-CphI and Endo VII residues are shown on top of the alignment.

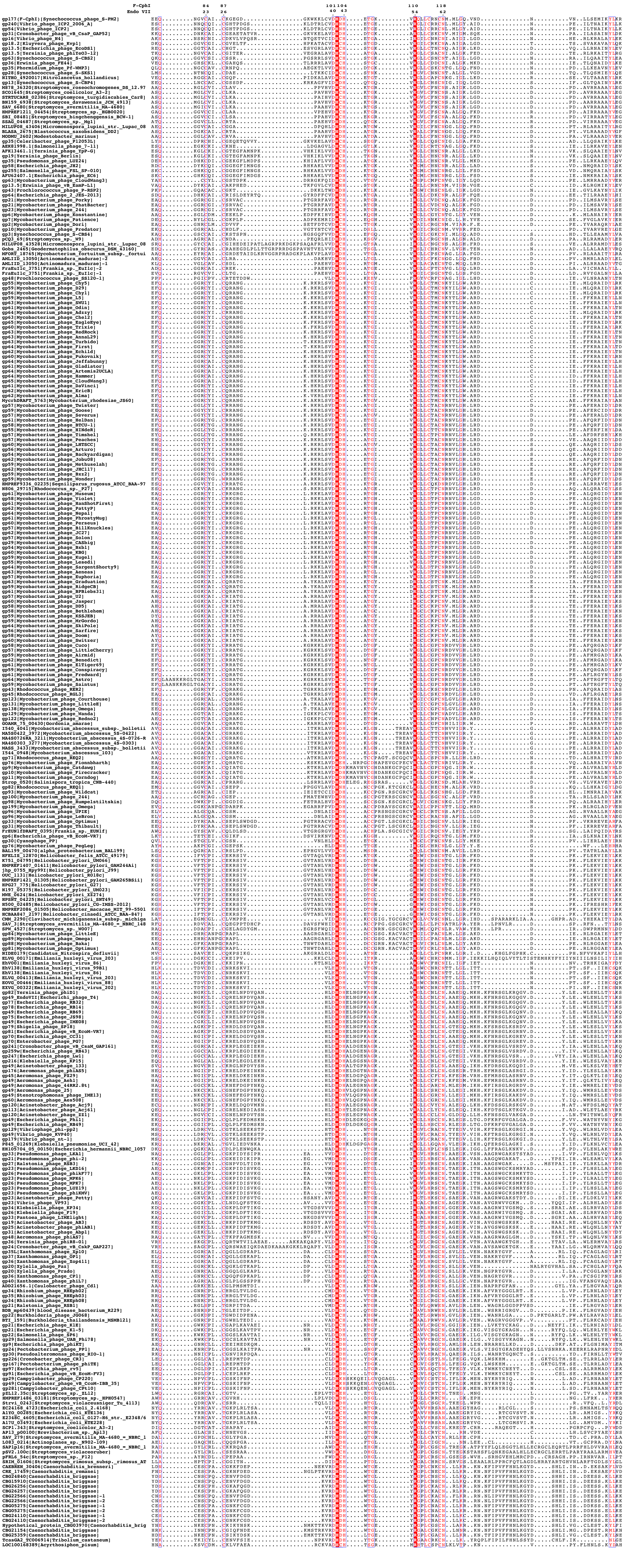

Supplement: Supplementary file 3 — Figure S2. Multiple sequence alignment of the Endo VII motif sequences. Dots represent gaps in the alignment. Totally conserved residues at a position are shown by white letters in red background. Residues with conservation above 70% at a position are shown by red letters in blue boxes. The position numbers of F-CphI and Endo VII residues are shown on top of the alignment. (PDF 1993 kb) [file 13100_2018_132_MOESM3_ESM.pdf]
