# Supplementary material for: F-CphI represents a new homing endonuclease family using the Endo VII catalytic motif
Source: Mob DNA. 2018 Aug 9;9:27. doi: 10.1186/s13100-018-0132-5 (PMC6083498; doi:10.1186/s13100-018-0132-5)
Supplement: Supplementary file 4 — Figure S3. Gel shift assay of F-CphI wild type and mutants. (A) 2 nM 32P labeled 60 bp duplex containing the F-CphI recognition site was incubated on ice for 15 min with increasing concentration of each protein. The free DNA (F) and protein-bound complexes (C) were separated on 8% native polyacrylamide gel. (B) 2 nM 32P labeled 60 bp duplex containing the F-CphI recognition site and 200 nM protein were incubated with increasing concentration of unlabeled non-specific 60 bp duplex (0 nM to 20 nM). The first lane in each gel shows the pattern of free DNA (no protein was added in the reaction). (C) Using the gels in A, the fractions of protein-bound complexes for wild type, D101N, and H102T were plotted against protein concentrations. The binding curves were generated from the non-linear regression fitted data, and were used to estimate the apparent equilibrium dissociation constant (Kd). Kd values are shown in each graph and errors represent 95% confidence interval. (PDF 126 kb) [file 13100_2018_132_MOESM4_ESM.pdf]

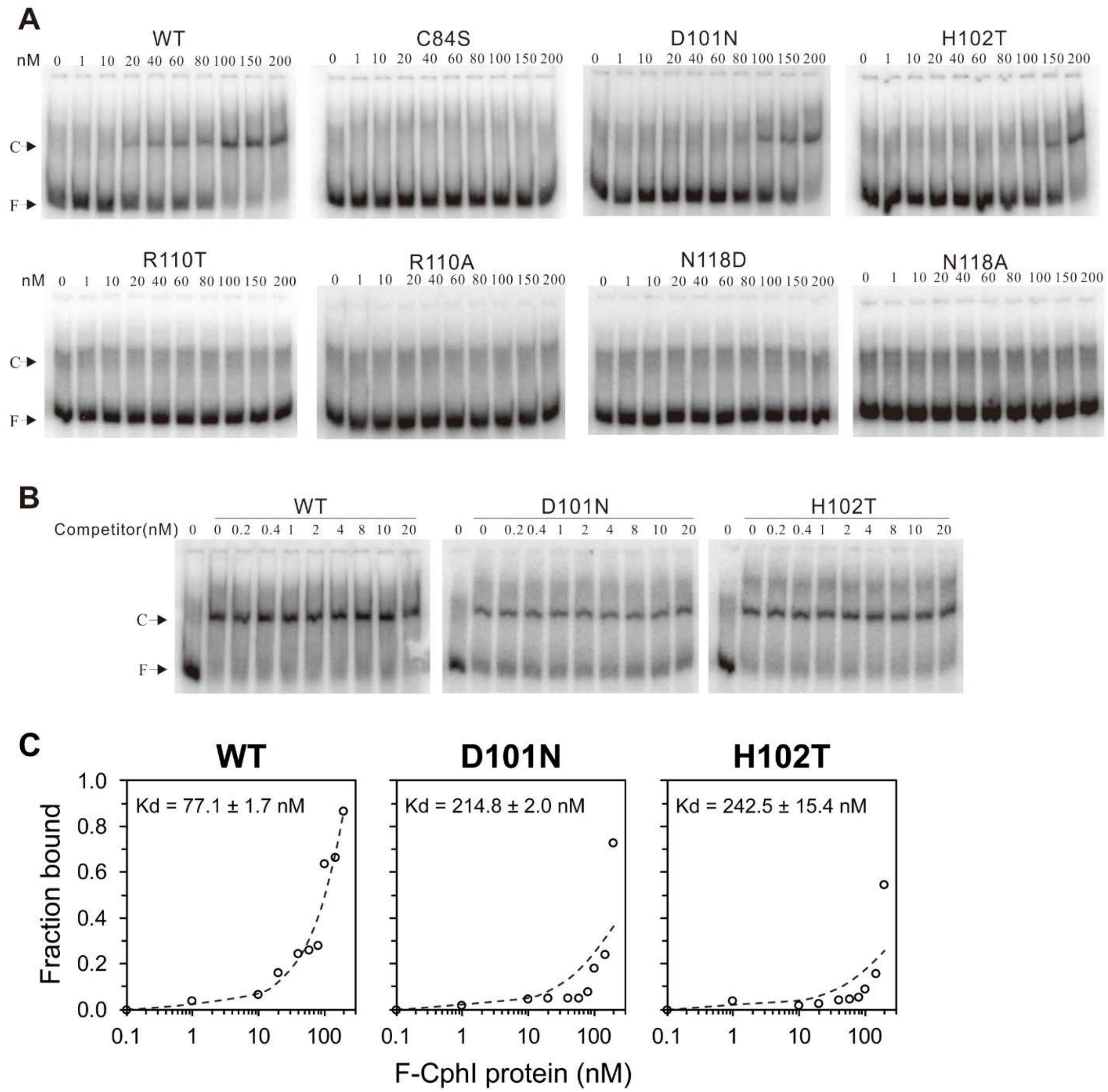

Supplementary Figure 3. Gel shift assay of F-CphI wild type and mutants

(**A**) 2 nM ^{32}P labeled 60 bp duplex containing the F-CphI recognition site was incubated on ice for 15 min with increasing concentration of each protein. The free DNA (F) and protein-bound complexes (C) were separated on 8% native polyacrylamide gel. (**B**) 2 nM ^{32}P labeled 60 bp duplex containing the F-CphI recognition site and 200 nM protein were incubated with increasing concentration of unlabeled non-specific 60 bp duplex (0 nM to 20 nM). The first lane in each gel shows the pattern of free DNA (no protein was added in the reaction). (**C**) Using the gels in **A**, the fractions of protein-bound complexes for wild type, D101N, and H102T were plotted against protein concentrations. The binding curves were generated from the non-linear regression fitted data, and were used to estimate the apparent equilibrium dissociation constant (Kd). Kd values are shown in each graph and errors represent 95% confidence interval.
