# Supplementary material for: F-CphI represents a new homing endonuclease family using the Endo VII catalytic motif
Source: Mob DNA. 2018 Aug 9;9:27. doi: 10.1186/s13100-018-0132-5 (PMC6083498; doi:10.1186/s13100-018-0132-5)
Supplement: Supplementary file 5 — Figure S4. Sequence logos of the DHHRN, HNH, and His-Cys box endonuclease families. Sequences near the active site of an endonuclease family are used to generate sequence logos. At each position of the sequence logo, the total height of a stack of letters shows the information content in bits that is calculated from a profile hidden Markov model, and the height of a letter relative to the total height of letters at a position represents the letter’s frequency. The red lines indicate gaps in the multiple sequence alignment. Above each sequence logo, the corresponding residue numbers of Endo VII, I-HmuI, and I-PpoI are shown, which are representatives of the DHHRN, HNH, and His-Cys box families, respectively. Black boxes show the corresponding catalytic residues used by Endo VII, I-HmuI, and I-PpoI. (PDF 351 kb) [file 13100_2018_132_MOESM5_ESM.pdf]

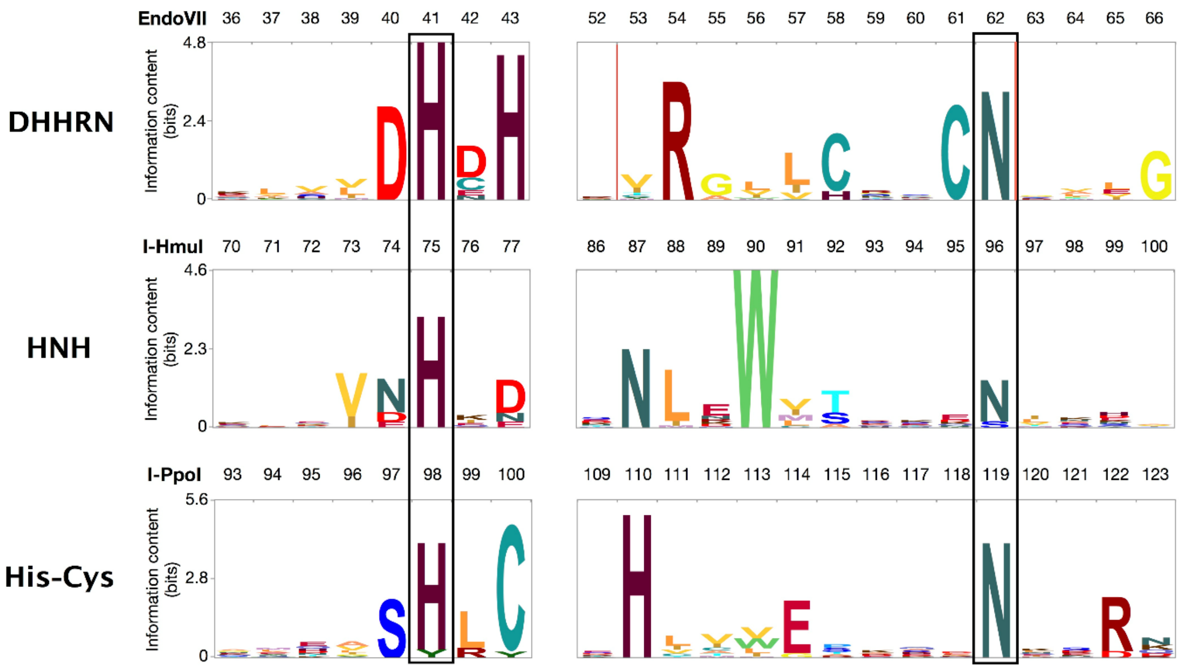

Supplementary Figure 4. Sequence logos of the DHHRN, HNH, and His-Cys box endonuclease families

Sequences near the active site of an endonuclease family are used to generate sequence logos. At each position of the sequence logo, the total height of a stack of letters shows the information content in bits that is calculated from a profile hidden Markov model, and the height of a letter relative to the total height of letters at a position represents the letter's frequency. The red lines indicate gaps in the multiple sequence alignment. Above each sequence logo, the corresponding residue numbers of Endo VII, I-HmuI, and I-PpoI are shown, which are representatives of the DHHRN, HNH, and His-Cys box families, respectively. Black boxes show the corresponding catalytic residues used by Endo VII, I-HmuI, and I-PpoI.
