# Supplementary material for: F-CphI represents a new homing endonuclease family using the Endo VII catalytic motif
Source: Mob DNA. 2018 Aug 9;9:27. doi: 10.1186/s13100-018-0132-5 (PMC6083498; doi:10.1186/s13100-018-0132-5)
Supplement: Supplementary file 6 — Table S3. Oligonucleotides used in this study (restriction sites are underlined and mutated sites are italicized). (DOCX 12 kb) [file 13100_2018_132_MOESM6_ESM.docx]

**Supplementary Table 3**. Oligonucleotides used in this study (restriction sites are underlined and mutated sites are italicized)

| **Name** | **Sequence (5’ to 3’)** |
| --- | --- |
| CyaU-Nco | AAACACTCATGACTAAACTATACTCTG |
| CyaD-Xba | GTTTTCTAGAGCTTGATACCTCTTTAAGTATTTAATCAT |
| SBM4-60Top | TGGTCTGGGTATGGAAGTGATGCACGAGCGCAACGCTCACAACTTCCCTC TCGACCTTGC |
| SBM4-60Top-r | complement of SBM4-60Top |
| endoV41A | TAGACGGATGAATATTGAGGACAGAAGTTGGATTTGGTAGT |
| endoV41comp | complement of endoV41A |
| C84S | cagaatggagtt*a*gtgctatttgta |
| C84Sr | tacaaatagcac*t*aactccattctg |
| D101N | aaactttgcgta*a*a*c*catgaccatg |
| D101Nr | catggtcatg*g*t*t*tacgcaaagttt |
| H102T | ctttgcgtagat*ac*tgaccatgaaac |
| H102Tr | gtttcatggtca*gt*atctacgcaaag |
| D103N | gcgtagatcat*a*accatgaaactg |
| D103Nr | cagtttcatggt*t*atgatctacgc |
| H104T | gtagatcatgac*ac*tgaaactggtaa |
| H104Tr | ttaccagtttca*gt*gtcatgatctac |
| R110A | actggtaaagtt*gc*tcagttgctttg |
| R110Ar | caaagcaactga*gc*aactttaccagt |
| N118D | gtaggaactgc*g*atatgatgttgg |
| N118Dr | ccaacatcatat*c*gcagttcctac |
| N118A | GTAGGAACTGC*GC*TATGATGTTGG |
| N118Ar | CCAACATCATA*GC*GCAGTTCCTAC |
